# Learning Representations from Heart Sound: A Comparative Study on Shallow and Deep Models

**DOI:** 10.34133/cbsystems.0075

**Published:** 2024-03-04

**Authors:** Kun Qian, Zhihao Bao, Zhonghao Zhao, Tomoya Koike, Fengquan Dong, Maximilian Schmitt, Qunxi Dong, Jian Shen, Weipeng Jiang, Yajuan Jiang, Bo Dong, Zhenyu Dai, Bin Hu, Björn W. Schuller, Yoshiharu Yamamoto

**Affiliations:** ^1^ Key Laboratory of Brain Health Intelligent Evaluation and Intervention, Ministry of Education (Beijing Institute of Technology), Beijing 100081, China.; ^2^ School of Medical Technology, Beijing Institute of Technology, Beijing 100081, China.; ^3^ Educational Physiology Laboratory, Graduate School of Education, The University of Tokyo, Tokyo 113-0033, Japan.; ^4^ Department of Cardiology, Shenzhen University General Hospital, Shenzhen 518055, China.; ^5^ CHI – Chair of Health Informatics, Technical University of Munich, Munich 81675, Germany.; ^6^ Department of Cardiovascular, Wenzhou Medical University First Affiliated Hospital, Wenzhou 325000, China.; ^7^ GLAM – Group on Language, Audio & Music, Imperial College London, London SW7 2AZ, UK.

## Abstract

Leveraging the power of artificial intelligence to facilitate an automatic analysis and monitoring of heart sounds has increasingly attracted tremendous efforts in the past decade. Nevertheless, lacking on standard open-access database made it difficult to maintain a sustainable and comparable research before the first release of the PhysioNet CinC Challenge Dataset. However, inconsistent standards on data collection, annotation, and partition are still restraining a fair and efficient comparison between different works. To this line, we introduced and benchmarked a first version of the Heart Sounds Shenzhen (HSS) corpus. Motivated and inspired by the previous works based on HSS, we redefined the tasks and make a comprehensive investigation on shallow and deep models in this study. First, we segmented the heart sound recording into shorter recordings (10 s), which makes it more similar to the human auscultation case. Second, we redefined the classification tasks. Besides using the 3 class categories (normal, moderate, and mild/severe) adopted in HSS, we added a binary classification task in this study, i.e., normal and abnormal. In this work, we provided detailed benchmarks based on both the classic machine learning and the state-of-the-art deep learning technologies, which are reproducible by using open-source toolkits. Last but not least, we analyzed the feature contributions of best performance achieved by the benchmark to make the results more convincing and interpretable.

## Introduction

Auscultation, as a simple, convenient, cheap, noninvasive, and efficient clinical method, has been used by physicians over a century [1]. Accurately monitoring and understanding the heart sounds can be essential for the early-stage diagnosis and proper management of the cardiovascular diseases (CVDs), which are ranked as the first leading cause of death globally [2–4]. However, training professional medical interns who can make an efficient use of their stethoscopes is not an easy task [5]. Within the fast development of machine learning (ML) and its cutting-edge subset, deep learning (DL), automatic analyzing and monitoring of the heart sounds has increasingly attracted numerous efforts from the community of both the medical and engineering fields [1,6]. Due to the noninvasive characteristic in nature, heart sound classification can be a feasible and efficient way for not only the early cost-effective screening of CVDs but also managing the progression of its condition [1]. Furthermore, this study can benefit the Internet of Things-based assisted living [7], personalized healthcare [8], and smart home monitoring systems [9].

Nevertheless, it maintained a challenge in a long time for researchers to train and validate their automatic heart sound classification algorithms due to a lack of high-quality, rigorously validated, and standardized open-access databases of heart sound recordings [6]. To address this challenge, Liu et al. released the 2016 PhysioNet/Computing in Cardiology (CinC) Challenge heart sound recordings as the first large open-access standard heart sound database [10]. Besides, the authors provided an open-source toolkit for implementing automatic segmentation and classification of the heart sounds. On one hand, the CinC database dramatically promoted and encouraged scientific community to research and develop algorithms for heart sound classification task, which included both the classic ML methods needing human hand-crafted features and state-of-the-art DL techniques that can learn higher representations from the raw signal itself [6,10]. On the other hand, there are still some limitations existing in the CinC database: First, multicenter data collection (from 8 different sources) makes CinC inconsistent in the data acquisition system, environment, and annotation process. This may arise issues to make an intelligent model without any external uncertainties or interference. Second, the CinC database ignored a subject-independent data partition, which may result in an overoptimistic evaluation of the final performances. Third, more reasonable evaluation metrics for the imbalanced data set, e.g., unweighted average recall (UAR), were not used in CinC Challenge. To this line, we proposed our first version of the open-access standard subject-independent heart sound database, i.e., the Heart Sounds Shenzhen (HSS) corpus [11]. In [11], a basic comparison between classic ML models (SVM) and DL models (long short-term memory [LSTM]/gated recurrent unit [GRU]-RNN) was investigated. However, human hand-crafted features were used in that study, which cannot give the audience a view of using state-of-the-art techniques that can learn high-level features automatically from the heart sound via DL. In fact, we can see some recently published literature giving some encouraging results showing the trend on learning heart sound features in an unsupervised learning paradigm. However, a comprehensive study on the state-of-art representation learning paradigms on heart sound classification task is lacking. To this end, we introduce this work includes transfer learning, sequence-to-sequence learning and end-to-end learning approaches for the heart sound classification. For the reason that the relationship of the models usually is not clear, we utilize the Shapley values [12,13] to evaluate the global features contributions. To the best of our knowledge, this is the first time to present the comprehensive investigation on heart sound classification task.

The main updates and contributions of this work are: Firstly, we use a shorter duration (10 s) of the heart sound recordings, which is more similar to real human auscultation scenario in clinical practice. Secondly, besides the same task as in HSS, we add another subtask for classifying normal/abnormal heart sounds. We think this binary classification study can be important for fast early clinical screening or in-home monitoring of the subjects who are suffering from chronic CVDs. Thirdly, we present the benchmarks of both the classic ML models and the state-of-the-art DL paradigms for heart sound classification task. Moreover, the interpretation experiments of the best benchmark are given to examine the features importance. In addition, all the results are reproducible based on our open-source toolkits. Last but not least, we hope this study cannot only benefit the study on automatic heart sound classification, but also facilitate other domains using the cutting-edge machine listening techniques for healthcare or social wellbeing applications.

The remainder of the work will be organized as follows: the background and related work are introduced in Background and Related Work. Then the proposed database and tasks will be described in Database and tasks. In Classic ML models, we introduce the methods and toolkits used in this study in details. The experimental results will be presented in Results, and followed by a discussion in Discussion. Finally, we make a conclusion of this article in Conclusion.

**Fig. 1.**
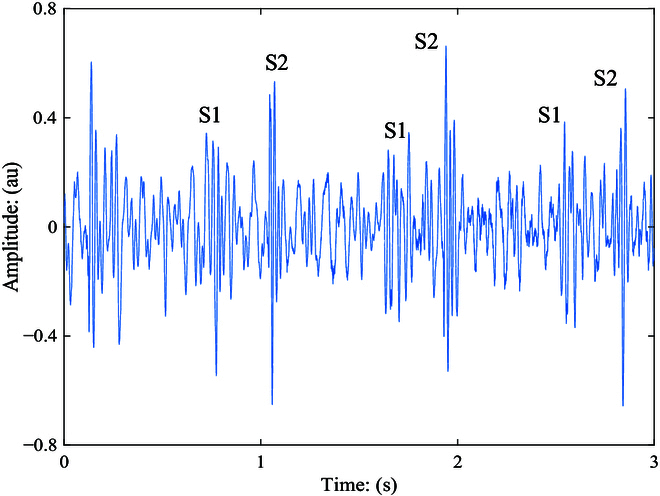
The normalized time waveform of the PCG (heart sound) signal. The fundamental components include the S1 (caused by the closure of the mitral and the tricuspid valves) and the S2 (caused by the closure of the aortic and the pulmonary valves). au, arbitrary unit.

The heart sound, a.k.a. phonocardiogram (PCG), has been studied and investigated to be a potential marker for both personal identification [14] and CVDs diagnosis [1] in tremendous prior works. As a noninvasive and inexpensive method by nature (cf. [15]), automatic computer-assisted analysis of the PCG signals cannot only substantially improve the diagnosis accuracy of the CVDs [16] but also avoid a cumbersome and expensive check by the Echocardiography approach [17]. In early works [1], classic ML models needing human hand-crafted features, e.g., Mel-frequency cepstral coefficients (MFCCs) [18], were used to help building ML models, e.g., support vector machine (SVM) [19], mapping the heart sound signals to the targeting predictions, e.g., normal/abnormal clips. In particular, the whole heart sound recordings should be firstly segmented into fundamental components [20] (see Fig. 1). In addition, in this scenario, electrocardiography is usually used as an auxiliary signal, which can identify the locations of the fundamental components in the cardiac cycle [1]. Nevertheless, as indicated by Dwivedi et al. [1], such kind of method has several disadvantages on requiring a secondary signal, difficulties in sensing and synchronization, depending on pathological conditions of subjects, and complex in computation and processing. Therefore, one recent trend of the relevant studies is to build a holistic automated system for diagnosis of CVDs via the PCG signals without any segmentation step [11].

Moreover, the ever-changing paradigms of ML, specifically for its hottest subset, DL, can learn higher representations from the data itself. On one hand, we are encouraged to see the state-of-the-art approaches like sequence-to-sequence learning [21] and transfer learning [22] are implemented successfully in the heart sound classification task, which can get rid of time-consuming and expensive human-designed features. On the other hand, the available standard open-access heart sound database is still insufficient. This obstacle not only restrains the power of DL models to learn robust and efficient representations but also makes it difficult to develop a reproducible and sustainable research in the field. To this end, we proposed our first version open-access heart sound database, i.e., HSS [11,23]. To the best of our knowledge, HSS is the current largest open-access heart sound database collected from a single medical center using consistent methods of data collection and annotation. A brief summary of the published literature based on HSS can be found in [11]. In this updated database, we use a 10-s-based long clip as the instance of heart sound, which is shorter than HSS (30 s). We may think that, this shorter duration makes it more challenging to learn sufficient information inherited in the heart sounds, for both machines and humans. In fact, in clinical practice, physicians usually perform the auscultation in one check for lasting approximately 10 s [24]. Additionally, apart from the same 3-class classification task in HSS [11], we add a binary classification task in this study. In this scenario, normal or abnormal heart sounds need to be classified, which can be crucial for both the prescreening in clinical practice and the in-home monitoring for subjects who are suffering from long-term chronic CVDs [25].

## Database and Tasks

In this section, we give the information of the database we proposed. In addition, we define the tasks needed to be addressed. This study was approved by the ethics committee of the Shenzhen University General Hospital, Shenzhen, P. R. China. All the participants agreed to use their data for research purposes.

### Database

**Fig. 2.**
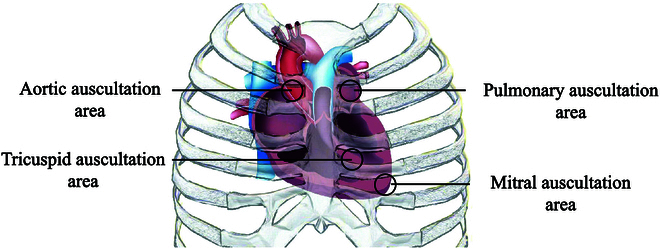
The 4 auscultation positions of the heart: aortic, pulmonary, tricuspid, and mitral.

The database is based on the whole original data in HSS [11] but having shorter clips of shorter duration. All of the original audio recordings in HSS were segmented into 10-s-based long clips with 5-s neighboring overlaps. Totally, 170 subjects (female: 55, male: 115, age: 65.4±13.2 years.) participated in the data collection. These subjects were with a variety of health conditions including hypertension, hyperthyroid, arrhythmia, coronary heart disease, heart failure, valvular heart disease, and congenital heart disease amongst others. All the heart sound audio recordings were recorded via an electronic stethoscope (Eko CORE, USA, Bluetooth 4.0, 4-kHz sampling rate) from 4 locations of the body (see Fig. 2), i.e., the auscultatory mitral, the aortic valve auscultation, the auscultatory areas of the tricuspid valve, and the pulmonary valve auscultation.

**Fig. 3.**
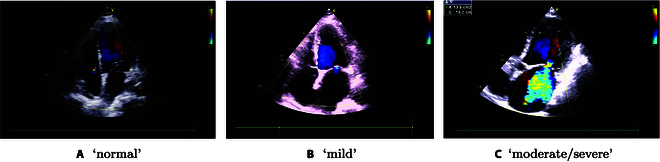
The echocardiography samples of the participants. (A) There were signals of regurgitation detected of the mitral valve orifice from the apical 4-chamber section. (B) There were color Doppler and a small amount of mitral regurgitation signals detected from the apical 4-chamber section. (C) There were mitral valve orifice and a lot of regurgitation signals detected from the apical 5-chamber section.

### Tasks

In this work, we have 2 subtasks, i.e., Task 1: Classification of normal, mild, and moderate/severe heart sounds (see Fig. 3); Task 2: Classification of normal, and abnormal (which includes the labels of mild or moderate/severe) heart sounds. In this study, the data partition are the same as in HSS [11], which is under the subject-independent condition. The details of the data partition can be found in Table 1.

**Table 1.** The number [#] of instances in data partition in this study

(A) Task 1				
	Train	Dev	Test	Σ
Normal	420	160	140	720
Mild	1,380	490	455	2,325
Moderate/Severe	710	250	220	1,180
Total	2,510	900	815	4,225
(B) Task 2				
	Train	Dev	Test	Σ
Normal	420	160	140	720
Abnormal	2,090	740	675	3,505
Total	2,510	900	815	4,225

## Methods and Toolkits

In this section, we give details of the methods used in this study. Besides, the open-source toolkits will also be introduced to reproduce this research.

### Classic ML models

In the classic ML paradigm, human hand-crafted features, as the front-end, expert’s domain knowledge are crucial for further model building. At the first step, low-level descriptors (LLDs) will be extracted from the audio signals (i.e., heart sounds in this study). Subsequently, supra*-*segmental features [26] summarizing statistical information can be obtained from the LLDs over a given period of the signals. In this study, we use the ComParE feature set LLDs (see Table 2) in our popular toolkit openSMILE. This standard feature set (including temporal and spectral acoustical properties) has been used successfully in the previous editions of the ComParE challenges starting from 2013 [27].

**Table 2.** The LLDs for ComParE feature set. The details can be found in [26].

4 Energy-related LLDs	Group
RMSE, zero-crossing rate	Prosodic
Sum of auditory spectrum (loudness)	Prosodic
Sum of RASTA-filtered auditory spectrum	Prosodic
6 Voicing-related LLDs	Group
*F*_0_ (SHS and Viterbi smoothing)	Prosodic
Prob. of voicing	Voice Quality
log HNR, jitter (local and *δ*), shimmer (local)	Voice Quality
55 Spectral LLDs	Group
MFCCs 1–14	Cepstral
Psychoacoustic sharpness, harmonicity	Spectral
RASTA-filt. aud. spect. bds. 1–26 (0–8 kHz)	Spectral
Spectral energy 250–650 Hz, 1 k–4 kHz	Spectral
Spectral flux, centroid, entropy, slope	Spectral
Spectral Roll-Off Pt. 0.25, 0.5, 0.75, 0.9	Spectral
Spectral variance, skewness, kurtosis	Spectral

RASTA, relative spectral transform; HNR, harmonics-to-noise ratio; RMSE, root mean square energy.

**Table 3.** The functionals applied to LLDs in the ComParE feature set. Note that, the LLDs listed in Table 2 may or may not use all of the functionals of this table, which is described in details in [26].

Functionals
Arithmetic or positive arithmetic mean
Root-quadratic mean, flatness
Standard deviation, skewness, kurtosis, quartiles 1–3
Inter-quartile ranges 1–2, 2–3, 1–3,
99-th and 1-st percentile, range of these
Relative position of max. and min. value
Range (difference between max. and min. values)
Linear regression slope, offset
Linear regression quadratic error
Quadratic regression coefficients
Quadratic regression quadratic error
Temporal centroid
Peak mean value and distance to arithmetic mean
Mean and std. dev. of peak to peak distances
Peak and valley range (absolute and relative)
Peak-valley-peak slopes mean and std. dev.
Segment length mean, min., max., std. dev.
Up-level time 25%, 50%, 75%, 90%
Rise time, left curvature time
Linear Prediction gain and coefficients 1–5

For the back-end, we use the popular SVM model [19], for its stable and efficient performance in the previous study [11]. For the implementation of SVM, we select the open-source toolkit LIBSVM [28].

#### Statistical functionals

**Fig. 4.**
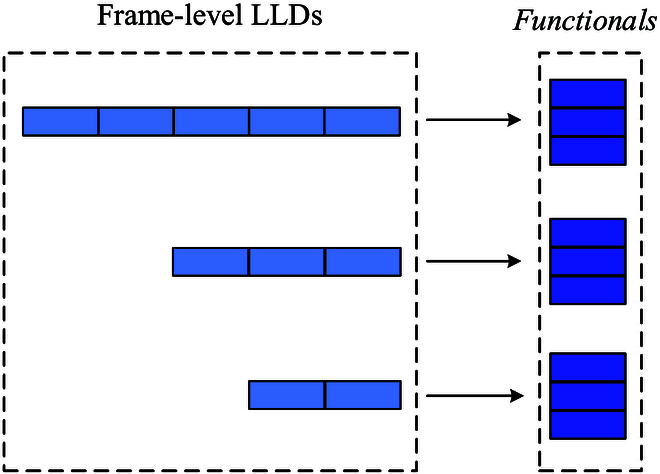
The diagram of the statistical functionals approach. We calculated a series of statistical functionals (e.g., max. min. mean, etc.) from the frame-level LLDs (e.g., MFCCs) extracted from the heart sound signal, which can be independent of the length of the instances.

The statistical functionals (func.), containing the mean, standard deviation, extremes, etc., are calculated from a given period of one audio clip (see Fig. 4). In this study, we use the default func. configuration in the ComParE feature set (see Table 3), which results in 6,373 features by applying func. to the LLDs and their first delta values.

#### Bag-of-audio-words approach

**Fig. 5.**
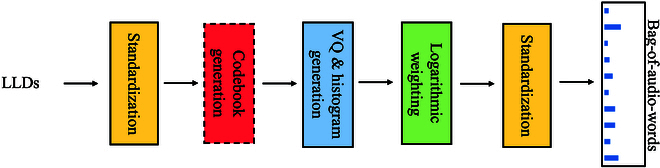
The diagram of the processing chain of the BoAW approach. The term frequency histograms are regarded as the representations extracted from the LLDs for further ML models.

The bag-of-audio-words (BoAW) approach was derived from the concept of bag-of-words (BoW, cf. [29]), which has been successfully applied in the domain of natural language processing [30] and computer vision [31,32]. In our proposed BoAW approach (see Fig. 5), a codebook was generated from the acoustic LLDs/deltas via as seeded random sampling process following the initialization step of k-means++ clustering [33]. When calculating the histograms, each LLD/delta is assigned to the 10 audio words from the codebook having the lowest Euclidean distance. In this study, both BoAW representations from the LLDs and their deltas are concatenated. We use a logarithmic term frequency weighting to compress the numeric range of the resulting histograms. The LLDs and their deltas are extracted using the openSMILE toolkit [34] with the ComParE feature set. The BoAW approach is implemented by the openXBOW toolkit [35]. For optimizing the codebook size *N_c_*, we investigate 125, 250, 500, 1,000, and 2,000 in this study.

**Fig. 6.**
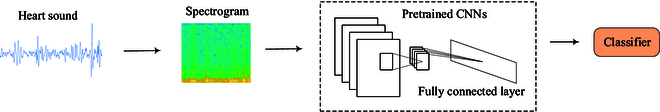
The diagram of the deep spectrum transfer learning approach. In this paradigm, we use a pretrained deep CNN model (e.g., AlexNet) to extract higher representations from the spectrograms transformed from the heart sounds. Then, a classifier (e.g., SVM) can make predictions based on the extracted representations.

### DL models

DL [36], which can extract higher representations from the data by using a series of nonlinear transformations of the inputs, is dramatically changing the paradigms of ML. In particular, compared with the classic ML methods (shallow models), DL models can learn more robust and generalized features when the data size becomes big. In this study, we propose 3 typical DL methods, i.e., the deep spectrum transfer learning method (see Fig. 6) by using pretrained DL models [22], recurrent sequence-to-sequence autoencoders (S2SAE) [21], and end-to-end (E2E) learning models [37].

#### Deep spectrum transfer learning

In this method, heart sound signals are fistly transformed into Mel-spectrograms (128 Mel frequency bands are computed) using a Hanning window with 32-ms width and 16-ms overlap. Then, the generated spectrograms are forwarded through a pretrained deep convolutional neural networks (CNN) [38]. Finally, the activations of the “avg_pool” later of the network are extracted as the higher representations for building the ML model (SVM in this study). For the pretrained CNNs, we investigate ResNet 50 [39], VGG 16 [40], VGG 19 [40], AlexNet [41], and GoogLeNet [42].

#### Recurrent S2SAEs

**Fig. 7.**
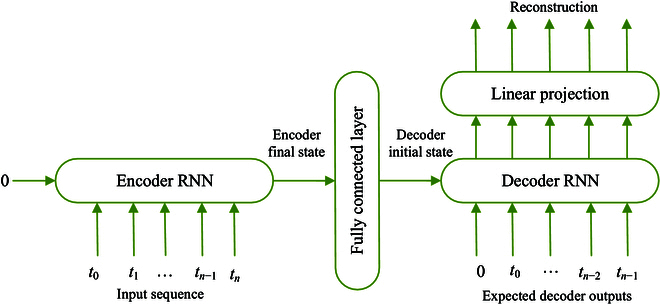
The diagram of the recurrent autoencoder based S2SAE approach. In this approach, an unsupervised scenario is used to learn higher representations. The network is trained to minimize the root mean square errors between the input sequence and the reconstruction. When the training is complete, we regard the activations of the fully connected layer as the representations of the input sequence.

In this approach, the first step is the same as in the aforementioned deep spectrum transfer learning method, namely, Mel-scale spectrograms are generated from the raw heart sound data. In addition, power levels are clipped below certain predefined thresholds in those spectrograms to eliminate some background noise (in this study, power levels are clipped below 4 different given thresholds, i.e., −30, −45, −60, and −75 dB). Then, in an unsupervised scenario, i.e., without any labels, a distinct recurrent S2SAE is trained on each of those sets of spectrograms. Finally, the learnt representations of a spectrogram are concatenated to be the feature vector for the corresponding instance. We use the auDeep toolkit [43] to implement the S2SAE method (see Fig. 7) in this study.

#### End-to-end learning

**Fig. 8.**
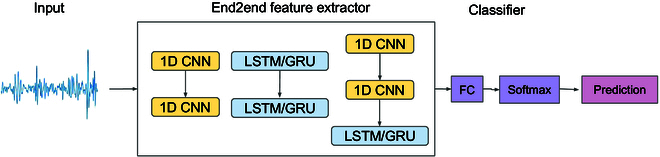
The diagram of the end-to-end learning approach. The higher representations can be extracted directly from the raw heart sound time waveforms with a series of deep CNN and/or RNN models.

The E2E model utilizes a series of CNNs [38] and/or recurrent neural networks (RNNs) [44] to extract higher representations directly from the raw heart sound audio waveforms (see Fig. 8). The previous studies had achieved success by the E2E models in analysis of music [45], speech emotion [46], and snore sound [37]. In this study, we use the deepSELF toolkit [47] for the E2E model implementation. To overcome the vanishing gradient problem in RNN training [48], we use the LSTM [49] and the GRU [50] cells when building the deep RNN models.

### Evaluation metrics

We use the UAR as the main evaluation metrics in this study by taking the data imbalanced characteristic into account. Compared to the widely used weighted average recall (WAR), i.e., the accuracy, UAR is more reasonable and rigorous for imbalanced database [51]. It is defined as:

$$\mathrm{UAR}=\frac{\sum_{i=1}^{N_{\text{class}}}{\text{Recall}}_{i}}{N_{\text{class}}},$$ (1)

where *Recall_i_* and *N_class_* are the recall of the *i*-th class and the number of classes, respectively. The WAR (accuracy) can defined as:

$$\begin{array}{l} \mathrm{WAR}=\sum_{i=1}^{N_{\text{class}}}\lambda_{i}{\text{Recall}}_{i},\\ \lambda_{i}=\frac{N_{i}}{N}, \end{array}$$ (2)

where *λ_i_* is called the weight for the *i*-th class, *N_i_* is the total number of instances labeled as the *i*-th class, *N* is the total number of instances.

To evaluate the performances of the models for Task 2 (the binary classification task), we use accuracy (acc., cf. Eq. 2), sensitivity (sens.), specificity (spec.), precision (prec.), F1-score, and G-mean values as complementary metrics. We have the definitions as:

$$\text{sens}.=\frac{\mathrm{TP}}{\mathrm{TP}+\mathrm{FN}},$$ (3a)

$$\text{spec}.=\frac{\mathrm{TN}}{\mathrm{TN}+\mathrm{FP}},$$ (3b)

$$\text{prec}.=\frac{\mathrm{TP}}{\mathrm{TP}+\mathrm{FP}},$$ (3c)

$$F1-\text{score}.=2\cdot \frac{\text{prec}.\times \;\text{recall}}{\text{prec}.+\;\text{recall}},$$ (3d)

$$G-\text{mean}=\sqrt{\text{sens}.\times \;\text{spec}.},$$ (3e)

where recall is the same as sens., TP, TN, FP, and FN are the number of true positive (“Abnormal” correctly identified as “Abnormal”), true negative (“Normal” correctly identified as “Normal”), false positive (“Normal” incorrectly identified as “Abnormal”), false negative (“Abnormal” incorrectly identified as “Normal”), respectively.

In addition, when comparing 2 results, we use the significance level test by one-tailed *z*-test [52]. The results can be regarded as significant when the *P* value is larger than 0.05.

### Explainable method SHAP

In many applications, the importance of understanding why a model makes a particular prediction is just as significant as the accuracy of the prediction itself. To interpret the contribution of the each feature to the prediction, we utilize SHAP (SHapley Additive exPlanations) [12] to explain how the features affect the predictions. The Shapley values is defined as follows:

$$g\left(z^{\prime }\right)=\phi_{0}+\sum_{i=1}^{M}\phi_{i}{z_{i}}^{\prime }$$ (4)

where *g*(*z*′) is the explanation model, *ϕ*_0_ is a constant (usually the mean value of the target variable for all samples), *ϕ_i_* is the Shapley value of the feature *i*, $z_{j}^{\prime }\in {\left\{0,,,1\right\}}^{M}$, and *M* is the number of the input features.

Contrasting to the traditional feature importance, the Shapley value can be “positive” or “negative”. When *ϕ_i_* > 0, it means the feature *i* improve the prediction of the model, i.e., positive. Conversely, it indicates that the feature *i* leads to a decrease in the predicted value, existing a negative effect. In addition, the greatest advantage of SHAP is that it is able to reflect the influence of the features in each sample.

## Experimental Results

In this section, we describe the experimental settings at first. Then, we show the results achieved by this work.

### Setup

All the experiments in this study are run by Python-based scripts. For reproducibility, we use opensource toolkits to implement the 5 methods aforementioned. For the SVM model, we use the Python sklearn toolkit (linear kernel is chosen), which is based on the LIBSVM toolkit [28]. [https://scikit-learn.org/stable/modules/generated/sklearn.svm.SVC.html.] All the hyper-parameters are tuned and optimized on the dev set, and applied to the test set. For showing results, the dev results are the ones achieved by the optimal models and the test results are the ones validated by the models within optimized hyper-parameters on the dev set. To minimize the effects by data imbalanced characteristic, we use upsampling technique to replicate the instances that belong to the scarce class. All the features are standardized before fed into the classifier by using the mean and standard deviation values from the train set.

### Results

The experimental results of the proposed 5 methods are listed in Table 4. We can see that, for both Task 1 and Task 2, the ComParE feature set-based models dominate the best performances. It is consistent with our previous studies [11,23], currently the well-designed human expert hand-crafted features are important for building an efficient and robust model for heart sound classification. For both Task 1 and Task 2, the best single models are all achieved by the classic ML model, i.e., the ComParE func. plus SVM classifier. The corresponding best UARs for the 3-class and binary class tasks are 48.8% (chance level: 33.3%) and 58.6% (chance level: 50.0%), respectively (chance level: the level that would be expected by random choices). These results are all significantly better than the E2E models in this study (*P* < 0.001, by one-tailed *z*-test). A late fusion (by majority vote) of the best 4 models reaches a comparable performance. For Task 1, the best fusion model yields to the best single model (UAR: 47.2% versus 48.8%). For Task 2, the best fusion model has a very slight improvement compared to the best single model (UAR: 58.7% versus 58.6%).

**Table 4.** Results for the benchmarks. *C*: Complexity parameter of the SVM. *N_c_*: Codebook size of bag-of-audio-words (BoAW) splitting the input into 2 codebooks (ComParE-LLDs/ComParE-LLD-Deltas), with 10 assignments per frame, optimized complexity parameter of SVM. *X*: Power levels which are clipped below 4 given thresholds. *N*_*E2E*_: Number of layers in LSTM/GRU/CNN models for end-to-end (E2E) learning. UAR: Unweighted Average Recall. Task 1: Three-Category (normal, mild, and moderate/severe) Classification (Chance Level: 33.3% of UAR); Task 2: Binary (normal and abnormal) Classification (Chance Level: 50.0% of UAR). The best results on the dev and test sets are highlighted in bold font. The best results on the test set are also marked with a gray background.

UAR [%]	Task 1		Task 2	
	Dev	Test	Dev	Test
*C*	openSMILE: ComParE func. + SVM			
10^−5^	43.7	43.7	61.6	55.2
10^−4^	**43.9**	**48.8**	**63.7**	**58.6**
10^−3^	41.8	45.2	57.6	57.2
10^−2^	42.3	42.4	57.7	54.1
10^−1^	41.6	40.4	58.7	52.0
1	40.8	40.0	57.9	52.9
*N_c_*	openXBOW: ComParE BoAW + SVM			
125	42.3	36.8	62.4	53.0
250	45.0	41.7	66.4	56.8
500	**45.9**	**46.9**	**68.6**	**58.5**
1,000	43.8	48.3	66.7	54.7
2,000	41.6	45.7	66.4	54.9
*X*	auDeep: RNN + SVM			
−30 *dB*	37.5	36.4	57.6	52.9
−45 *dB*	**39.7**	**40.2**	59.7	56.8
−60 *dB*	37.8	38.6	**60.8**	**55.4**
−75 *dB*	38.1	38.9	57.7	55.2
fused	38.9	37.8	58.5	54.7
Network	DeepSpectrum + SVM			
ResNet 50	**46.1**	**40.7**	**65.5**	**56.8**
VGG 16	42.4	42.0	63.3	53.9
VGG 19	39.8	42.4	59.1	54.2
AlexNet	42.4	40.9	62.1	51.5
GoogLeNet	41.9	44.3	64.3	53.8
Topology	deepSELF: E2E, *N*_*E2E *_= 2			
CNN	35.9	33.4	50.0	50.0
LSTM	**38.0**	**31.8**	54.5	48.7
GRU	36.3	32.6	54.8	52.4
CNN+LSTM	36.9	35.6	54.4	50.4
CNN+GRU	37.0	35.8	**57.1**	**48.3**
*n*	Fusion of *n*-Best			
3	–	48.1	–	58.7
4	–	**47.2**	–	**58.7**
5	–	44.6	–	58.0

The SHAP interpretation experiments are conducted on the all test set (815 samples) and we analyzed the feature contributions for the top 2 results in the baseline(i.e., ComParE 10^–4^ and ComParE 10^–3^). The SHAP explanation of the best performance achieved by the ComParE feature set is as shown in Fig. 10. From the figure, it can be seen that functional transformed feature MFCC and RASTA-filtered auditory spectrum have the highest feature contributions and features of the function of PeakMeanRel (relative mean value of the peak in a specific feature) usually get the better contributions.

Tables 5 and 6 show the confusion matrices of the best 4 models and their late fusion results on the test set for Task 1 and Task 2, respectively. For Task 1, the recall of the “Mild” and “Moderate/Severe” types of heart sounds are higher than the “Normal” ones. The fusion of the best 3 models can slightly improve the recall of the “Moderate/Severe” types compared with individual models whereas remains a low recall of the other 2 types of heart sounds. For Task 2, the recalls of the “Abnormal” types are much higher than the recalls of the “Normal” types. In other words, the “Normal” ones tend to be recognised as “Abnormal” ones. Fusion of the best 3 models can lead to a highest recall of the “Abnormal” types.

**Table 5.** The confusion matrices (normalized: in [%]) for Task 1 by the best models on the test set. N, Normal; M, Mild; S, Moderate/Severe. 1st Best Model: openSMILE: ComParE func. + SVM, *C*: 10^–4^; 2nd Best Model: openXBOW: ComParE BoAW + SVM, *N_c_*: 500, *C*: 10^–4^; 3rd Best Model: DeepSpectrum + SVM, Network: ResNet 50, *C*: 10^–4^; 4th Best Model: auDeep: RNN + SVM, *X* = −45 dB, *C*: 10^–1^ Fusion: A Late Fusion of the Best Four Models by Majority Vote.

(A) 1st Best Model
Pred ->	N	M	S
N	42.1	38.6	19.3
M	22.0	57.7	20.7
S	21.8	31.4	46.8
(B) 2nd Best Model
Pred ->	N	M	S
N	43.6	38.6	17.9
M	23.5	47.0	29.5
S	30.9	19.1	50.0
(C) 3rd Best Model
Pred ->	N	M	S
N	32.1	55.0	12.9
M	23.5	39.1	37.4
S	21.4	27.7	50.9
(D) 4th Best Model
Pred ->	N	M	S
N	32.9	35.7	31.4
M	25.5	42.6	31.9
S	18.6	36.4	45.0
(E) Fusion
Pred ->	N	M	S
N	39.3	45.0	15.7
M	24.0	47.9	28.1
S	20.9	24.5	54.5

**Table 6.** The confusion matrices (normalized: in [%]) for Task 2 by the best models on the test set. N: Normal; A: Abnormal. 1st Best Model: openSMILE: ComParE func. + SVM, *C*: 10^–4^; 2nd Best Model: openXBOW: ComParE BoAW + SVM, *N_c_*: 500, *C*: 10^–4^; 3rd Best Model: DeepSpectrum + SVM, Network: ResNet 50, *C*: 10^–4^; 4th Best Model: auDeep: RNN + SVM, *X* = −60 dB, *C*: 10^–2^. Fusion: A Late Fusion of the Best Four Models by Majority Vote.

(A) 1st Best Model		
*Pred* ->	N	A
**N**	45.7	54.3
**A**	28.4	71.6
(B) 2nd Best Model		
Pred ->	N	A
N	47.1	52.9
A	30.2	69.8
(C) 3rd Best Model		
Pred ->	N	A
N	42.1	57.9
A	28.4	71.6
(D) 4th Best Model		
Pred ->	N	A
N	41.4	58.6
A	30.7	69.3
(E) Fusion		
Pred ->	N	A
N	45.7	54.3
A	28.3	71.7

**Fig. 9.**
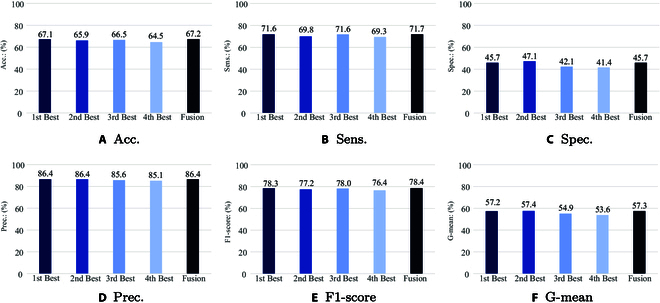
The complementary evaluation metrics (in [%]) achieved by the best models on the test set for Task 2. 1st Best Model: openSMILE: ComParE func. + SVM, *C*: 10^–4^; 2nd Best Model: openXBOW: ComParE BoAW + SVM, *N_c_*: 500, *C*: 10^–4^; 3rd Best Model: DeepSpectrum + SVM, Network: ResNet 50, *C*: 10^–4^; 4th Best Model: auDeep: RNN + SVM, *X* = −60 dB, *C*: 10^–2^. Fusion: A Late Fusion of the Best Four Models by Majority Vote.

Figure 9 illustrates the complementary metrics achieved by the best 4 models and their late fusion results on the test set for Task 2. It can be seen that, most of the models show higher results on acc., sens., prec., and F1-score while lower performances on spec. and G-mean. Fusion of the best 4 models cannot dramatically improve the single models’ performances.

**Fig. 10.**
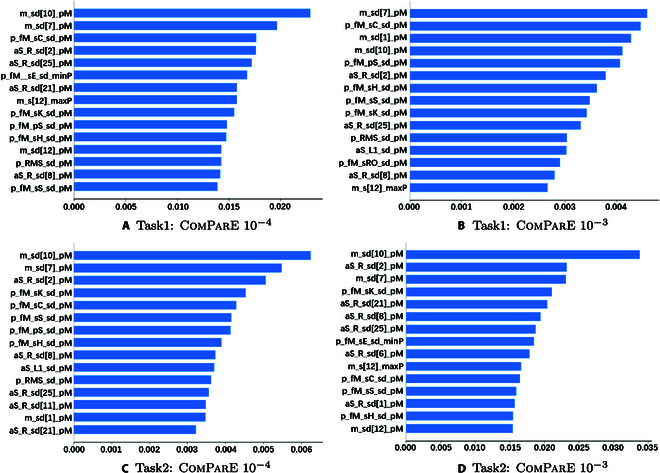
The SHAP explanation of the best performance achieved by the openSMILE: ComParE func. + SVM, *C*: 10^–4^ and 10^–3^; on the test set for Task 1 and Task 2. Meaning of abscissa: mean(∣SHAP value∣) (average impact on model output magnitude). m, mfcc; sd, sma_de; pM, peakMeanRel; p, pcm; fM, fftMag; sC, spectralCentroid; aS, audSpec; R, Rfilt; sE, spectralEntropy; minP, minPos; s, sma; maxP, maxPos; sK, spectralKurtosis; pS, psySharpness; sH, spectralHarmonicity; RMS, RMSenergy; sS, spectralSkewness; L1, lengthL1norm; sRO, spectralRollOff50.0.

## Discussion

In this section, we firstly indicate the current findings by this study. Then, we discuss the limitations from this work, and give our perspectives toward future work.

### Current findings

In this study, ComParE feature set based methods (func. and/or BoAW + SVM) are superior to other methods, in particular, for reaching the best results (see Table 4). We can see that, at current stage, finding efficient acoustical representations from the heart sound is the prerequisite. With the SHAP interpretation, we figure out that MFCC and RASTA-filtered auditory spectrum perform better in the heart sound classification.

In contrast, the performances of the 3 proposed DL based methods are modest. We segment audio clips into smaller duration length (10 s) compared with HSS 1.0 (around 30 s), which has increased the total number of instances from 845 in HSS 1.0 to 4,225 in this study. Nevertheless, DL models cannot generator better and more robust performances than the classic ML models. On one hand, we enjoy the benefits of DL methods getting rid of expensive, time-consuming, and inefficient human expert feature engineering process. On the other hand, we find it difficult to train a sufficiently robust and generalized DL model by using the current limited heart sound data resources.

Encouraging results can be found in acc., sens., prec., and F1-score. Specifically, the sensitivity (for detecting “Abnormal” heart sounds) can be higher than 70.0%, which can benefit a potential emergency care of subjects suffering from long-term chronic CVDs. However, the performances on spec. and G-mean are needed to be improved.

### Limitations and perspectives

The extreme data imbalance characteristic is still the first challenge for limiting the current performances of all the models. Even though the current best models can beat the chance level for both of the 2 tasks, the overall UARs are modest. In future work, we will continuously collect more “Normal” heart sounds, which can enrich the heart sound database. Additionally, we will involve more advanced technologies like generative adversarial networks [53], which were demonstrated to be efficient in the snore sound classification task [54].

Secondly, fundamental studies on heart sound feature analysis are lacking. The relationship between the acoustical properties and the anatomical changes in the heart under different kinds of CVDs is still unclear. Furthermore, more advanced signal processing should be investigated such as tunable-Q wavelet transformation [55], scaled spectrogram, and tensor decomposition [56].

Thirdly, we should overcome the big gap between the performances on the dev and the test sets. We think that, the subject-independency makes it difficult to reach high performances compared with other works based on subject-dependent data partition (e.g., the PhysioNet CinC Challenge Dataset [10]). This overfitting challenge should be overcome by developing more generalized models in future work.

Last but not least, more attentions and contributions should be attracted to this field. As a noninvasive method, heart sound analysis via ML methods have a promising potential in not only real clinical applications, but also in-home healthcare services. We will organize a series of open challenges and workshops in future to facilitate this research.

## Conclusion

In this study, we segmented the audio recordings in HSS into 10-s-based clips, which means an accurate prediction of heart status is needed from a shorter duration (around 30 s in HSS) of the audio recording. In addition, we added a binary classification task (normal/abnormal detection) as a subtask in this work. Both the classic ML and the cutting-edge DL methods were investigated and compared by using our open-source toolkits, which can be easily reproduced. In this benchmark study, the best result for the 3-class classification task was 48.8% of UAR (chance level: 33.3%). The best result for the binary classification task was 58.7% of UAR (chance level: 50.0%). Through the SHAP interpretation experiments, we analyzed the contribution of each feature in the entire test set and found that acoustic features MFCC and RASTA-filtered auditory spectrum performed the best in heart sound classification. We hope this new database and its benchmarks can further the relevant studies in a broad scientific community.

## Data Availability

The database will be provided by request only for research purpose.
